# Robust Adaptive Synchronization of Ring Configured Uncertain Chaotic FitzHugh–Nagumo Neurons under Direction-Dependent Coupling

**DOI:** 10.3389/fnbot.2018.00006

**Published:** 2018-02-26

**Authors:** Muhammad Iqbal, Muhammad Rehan, Keum-Shik Hong

**Affiliations:** ^1^Department of Computer and Information Sciences, Pakistan Institute of Engineering and Applied Sciences (PIEAS), Islamabad, Pakistan; ^2^Department of Electrical Engineering, Pakistan Institute of Engineering and Applied Sciences (PIEAS), Islamabad, Pakistan; ^3^Department of Cogno-Mechatronics Engineering, School of Mechanical Engineering, Pusan National University, Busan, South Korea

**Keywords:** FitzHugh–Nagumo neuron, neuronal networks, ring configuration, coupling strengths, robust adaptive synchronization control

## Abstract

This paper exploits the dynamical modeling, behavior analysis, and synchronization of a network of four different FitzHugh–Nagumo (FHN) neurons with unknown parameters linked in a ring configuration under direction-dependent coupling. The main purpose is to investigate a robust adaptive control law for the synchronization of uncertain and perturbed neurons, communicating in a medium of bidirectional coupling. The neurons are assumed to be different and interconnected in a ring structure. The strength of the gap junctions is taken to be different for each link in the network, owing to the inter-neuronal coupling medium properties. Robust adaptive control mechanism based on Lyapunov stability analysis is employed and theoretical criteria are derived to realize the synchronization of the network of four FHN neurons in a ring form with unknown parameters under direction-dependent coupling and disturbances. The proposed scheme for synchronization of dissimilar neurons, under external electrical stimuli, coupled in a ring communication topology, having all parameters unknown, and subject to directional coupling medium and perturbations, is addressed for the first time as per our knowledge. To demonstrate the efficacy of the proposed strategy, simulation results are provided.

## Introduction

The spurred efforts to get an insight of the complex and opaque interactions among the levels of various neuronal networks is a major aspiration in neuroscience, because it would be an incredible abet to explore the foundation of normal and pathological brain functioning (Buzsaki, 2006; Alvarellos-Gonzalez et al., 2012; Aqil et al., 2012b). For example, one would be able to unveil how a steering signal is generated for muscles from the brain or how neurons diminish during brain disorders like Parkinson’s, Huntington’s, and epilepsy (Deak et al., 2007; Di Garbo et al., 2007; Mejias and Torres, 2007; Limousin and Martinez-Torres, 2008; Jobst, 2010; and Ostrem and Starr, 2008). The brain’s mechanisms of operations have their own realism in interconnection and signal transmission, which has enthused many researchers to investigate brain activity at multiple levels (Naseer and Hong, 2013; Hong and Nguyen, 2014; Santosa et al., 2014; Hong and Naseer, 2016; Hong and Santosa, 2016; Nguyen and Hong, 2016; Zafar and Hong, 2017), ranging from a single neuron to a network of neurons. Brain has a number of complex functions and activities in relation to cognitive purposes (Santosa et al., 2013; Hong et al., 2015, 2017; Naseer et al., 2016; Nguyen et al., 2016). These brain activities can be somehow measured using various modalities and sensors in order to identify the intension of a subject (Turnip et al., 2011; Khan et al., 2014; Hong and Khan, 2017). Therefore, in-depth research has been done on modeling, analysis, instrumentation, and control of external devices in the area of brain-computer interfaces (Khan and Hong, 2015, 2017; Kocaturk et al., 2015; Naseer and Hong, 2015; Ghafoor et al., 2017; Liu and Hong, 2017).

Neuronal networks have been a thought-provoking and imperative subject owing to the various potential real-world processes, estimation, control and robotic applications [see Ellacott et al. (1997) and references therein]. In a neuronal network, a large number of neurons are inter-connected in various fashions under multifarious coupling phenomena. Recently, the studies on the dynamical behavior of a single neuron, a collective behavior of coupled neurons, and synchronization among the neurons have been extensively investigated (Thompson et al., 1999; Hua and Smith, 2004; Zhang et al., 2006; Wu and Chen, 2008; Yu et al., 2013; Wang et al., 2015). Synchronization of neurons plays a key role in the transmission process of neuronal signals, and enables effective communications in the brain or to the muscles (Knoblauch and Palm, 2005; Wang et al., 2008a,b; Nguyen and Hong, 2011, 2013). The FitzHugh–Nagumo (FHN) system, a simplified model of the coupling effect of neurons, has been considered largely owing to the fact that it mimics the dynamical behavior of neurons and intricates neuronal networks under external electrical stimulation (Thompson et al., 1999).

Neuroscience enriched by numerous reports in the context of coupled FHN neurons has opened a new avenue of research during the past few years. The simplest model to mimic the dynamical properties of neuronal interactions (such as synchronization) consists of two coupled neurons (Wang et al., 2009; Zhen and Xu, 2010; Aqil et al., 2012a; Iqbal et al., 2015, 2017). A control and synchronization methodology was designed to investigate the coupled reaction–diffusion FHN systems in Ambrosio and Aziz-Alaoui (2012). Synchronization of two coupled neurons was carried out by employing an adaptive backstepping sliding mode control in Yu et al. (2012). A theoretical criterion was presented for the synchronization of uncertain chaotic coupled systems for a neural network *via* the sliding mode technique by Chen et al. (2009). Synchronization of two identical coupled FHN systems with known or unknown parameters has been studied *via* a nonlinear adaptive control based on the fuzzy logic scheme, neural networks, the uncertainty estimator, and the feedback linearization control (Wang et al., 2007, 2008a,b; Zhang et al., 2007; Che et al., 2009), respectively. Later, a robust adaptive control for synchronization of two coupled FHN neurons of unknown parameters has been developed. Moreover, some important results for the synchronization of three-coupled FHN neurons having slightly different unknown parameters and disturbances with respect to multiple communication pathways have been explored (Rehan and Hong, 2011; Rehan et al., 2011). For more related investigations, synchronization of two coupled FHN neurons with unknown and different parameters under direction-dependent coupling has been discussed in Iqbal et al. (2014).

To a certain extent, efforts have been dedicated to the study of the dynamics of the neuronal networks coupled in a ring fashion, specifically by exploiting the impact of time delays (Campbell et al., 2005; Xu, 2008; Song and Xu, 2012; Zhang, 2014; Wang et al., 2015; Mao and Wang, 2016; Yuan et al., 2016; Mao, 2017). A recent work by Zhou et al. (2009) extended the synchronization problem to a network of coupled FHN neurons and explored the impact of the gap junctions on the network. It was investigated that the influence of the gap junctions on the dynamical behavior of neurobiological networks is stronger than the coupled systems. In addition, interestingly, a network of the FHN neurons exhibits a more fascinating dynamically complicated behavior than two or three coupled FHN neurons.

Some interesting works on synchronization of neurons have been accomplished in the recent years by employing various complexities. For instance, the work of Lai et al. (2008) employed an adaptive control approach, which provided synchronization of FHN neurons under a sinusoidal electrical field. The approach, however, may not ensure asymptotic convergence of the synchronization error and additional parameters are required for achieving the adaptation. To attain the robust synchronization of FHN neurons, Wei et al. (2009) introduced an internal model control strategy for output synchronization between the neurons using a semi-global Lyapunov approach. For dealing with perturbations, sliding surface-based control schemes were developed by Che et al. (2011) and Yu et al. (2012) in the presence of resistive coupling between the neurons. A step further, model complexity along with the behavioral analyses and control approach for phase synchronization between neurons were studied in the recent study by Ma et al. (2017). Despite of these studies, several open problems and challenges include synchronization in multiple coupled neurons and coupling model complexities.

In the earlier works, the research was limited to the simple scenarios of two or three coupled FHN neuronal models, since such simple scenarios were easily addressable. But, the operational mechanisms in the brain cannot be described with simple systems owing to the complex interactions (coupling) existing among the large number of neurons. Consequently, in order to explore the dynamical behavior of real complex systems, it is indispensable and challenging to work on larger coupled networks instead of a simple model of coupled systems (or reduced networks). In addition, the coupling models between the neurons should also be addressed as much as possibly closer to the actual complex medium strengths. Moreover, controlling of behaviors of neurons can be accomplished *via* adaptive control approaches in order to develop intelligent methods of adaptation according to the dynamical circumstances (Oyama et al., 2016; Stewart et al., 2016; Aoi et al., 2017). In conclusion, considering a neuronal network with unknown parameters in which a large number of neurons are communicating under complex couplings, namely, direction-dependent coupling, can lead to enhance the theoretical and numerical analysis of neuronal systems’ complexity, which is a pretty challenging research task.

Motivated by the aforementioned rationale, the aim of this paper is to investigate the dynamical behavior and synchronization of a network of different FHN neurons with unknown parameters, linked in a ring configuration, under direction-dependent coupling mediums. The direction-dependent coupling has been employed due to direction-dependent behavior of the gap junctions. The gap junctions between neurons can either allow current in one or in both (but with different strengths) directions, giving rise to the so-called direction-dependent coupling between neurons, see Iqbal et al. (2014). A model of four different FHN neurons, coupled in a ring topology, under external disturbances is presented. The different strength of the gap junctions for each link in the network owing to the inter-neuronal coupling medium properties is considered. A robust adaptive control is designed to address the intricate problem of the synchronization in a network of neurons. Based on Lyapunov stability theory, conditions are derived analytically for the synchronization in a network of four different FHN neurons with unknown parameters in a ring configuration under direction-dependent coupling and disturbances. The developed robust adaptive control algorithm encounters the problem of dealing with different recovery variables. Unlike the synchronization approach, partial synchronization of neurons by Iqbal et al. (2014), the proposed scheme ensures the complete synchronization of neurons. To the best of our knowledge, the robust adaptive control mechanism for synchronization of different neurons with unknown parameters in the ring configuration under direction-dependent coupling and disturbances is addressed for the first time. Essentially, the outcome of this study can edify new ideas for understanding of the neuronal networks in context of multifaceted coupling phenomena. Compared with the existing works on synchronization of two or three neurons, our study considers a complex scenario for synchronizing four neurons in a ring configuration under direction-dependent coupling, parametric uncertainties, and perturbations. This study shows the possibility of a robust and adaptive control strategy for attaining the coherent behavior among neurons forming a complicated network under an external electrical stimulation. To end with, extensive numerical simulation results are drawn to elucidate the efficacy of the proposed method.

There are several differences in this study compared to the existing works. For instance, this study considers a ring configuration of multiple neurons rather than an interconnection of two neurons as in Wang et al. (2007), Zhang et al. (2007), Wang et al. (2008a,b), Che et al. (2009), Rehan and Hong (2011), Lai et al. (2008), Wei et al. (2009), Che et al. (2011), Yu et al. (2012), and Ma et al. (2017). In addition, the current flow between two neurons is considered as direction-dependent, compared to these models, for regarding bidirectional coupling formed by the gap junctions. Moreover, the models of neurons in our study have different parameters to examine synchronization of dissimilar neurons. Compared to synchronization study in Rehan et al. (2011) for three FHN neurons, we develop a control approach for robust adaptive synchronization and all the parameters are considered to be unknown and different. Moreover, we employ a more complex scenario of four neurons, bidirectional coupling, and ring configuration. In comparison to the recent neuronal synchronization study of Iqbal et al. (2015), there are three contributions in this work. First, we consider multiple neurons for developing a synchronization control approach owing to the presence of multiple coupled neuronal interactions in the brain; second, synchronization of both activation potentials and recovery variables has been achieved in the proposed study; third, the idea of bidirectional coupling between two neurons has been extended to a ring configuration of neurons.

The rest of the manuscript are organized as follows: Section “Results and Discussion” discusses the main results, containing the modeling of a network of different FHN neurons with unknown parameters linked in a ring configuration under direction-dependent coupling, the design of a robust adaptive control mechanism, synchronization in the network without disturbance, synchronization in the network with disturbance, and numerical simulation results. Section “Methods” includes the employed methods, namely, FHN model, Lyapunov stability analysis, and proof of the main results without and with disturbances. Section “Conclusion”, finally, includes the study conclusions.

## Results and Discussion

### Ring Configured FHN Neurons under Direction-Dependent Coupling

**Figure 1:**
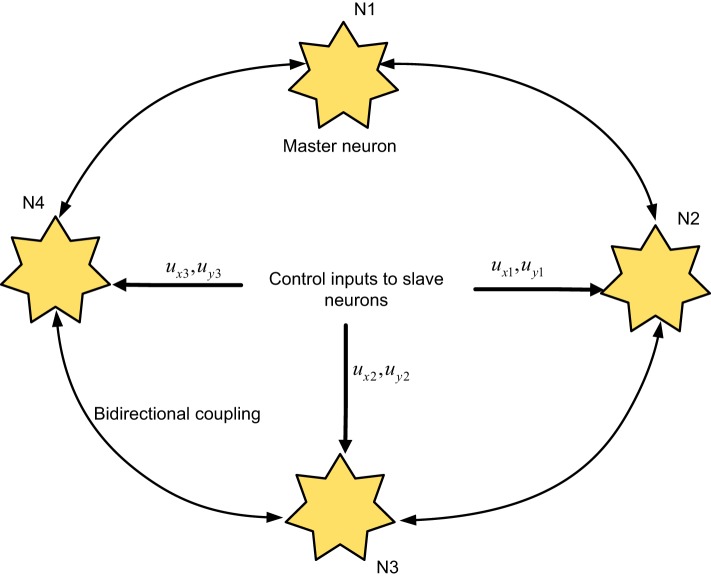
Four neurons in a ring configuration: the neurons are interconnected through bidirectional couplings; control inputs are used for synchronization of the slave neurons to the master neuron.

The ring configuration of four neurons coupled in a bidirectional medium is shown in Figure 1. Let N1 be the master neuron, and N2, N3, and N4 be the slave neurons. We employ control signals for the synchronization of the slave neurons with the master neuron. The purpose of this study is to model the neuronal behavior and to provide a synchronization control remedy for attaining the coherent behavior of the neurons. The proposed network model of ring configured four FHN neurons under direction-dependent coupling [by accounting the results of Iqbal et al. (2014) and Yuan et al. (2016)] is given by

(1) $$\begin{array}{lll} {\dot{x}}_{1}=x_{1}\left(x_{1}-1\right)\left(1-r_{1}x_{1}\right)-y_{1}-g_{1}\left[\;\left(x_{1}-x_{2}\right)+\left(x_{1}-x_{4}\right)\right]\; & & \;\\ \;+I_{\mathrm{ext},1}+d_{\mathrm{ext},1}, \end{array}$$

(2) $$\begin{array}{lll} {\dot{y}}_{1}=b_{1}x_{1},\; & \;\\ {\dot{x}}_{2}=x_{2}\left(x_{2}-1\right)\left(1-r_{2}x_{2}\right)-y_{2}-g_{2}\left[\;\left(x_{2}-x_{1}\right)+\left(x_{2}-x_{3}\right)\right]\; & & \;\\ \;+I_{\mathrm{ext},2}+d_{\mathrm{ext},2}, \end{array}$$

(3) $$\begin{array}{lll} {\dot{y}}_{2}=b_{2}x_{2},\; & \;\\ {\dot{x}}_{3}=x_{3}\left(x_{3}-1\right)\left(1-r_{3}x_{3}\right)-y_{3}-g_{3}\left[\;\left(x_{3}-x_{2}\right)+\left(x_{3}-x_{4}\right)\right]\; & & \;\\ \;+I_{\mathrm{ext},3}+d_{\mathrm{ext},3}, \end{array}$$

(4) $$\begin{array}{lll} {\dot{y}}_{3}=b_{3}x_{3},\; & & \;\\ {\dot{x}}_{4}=x_{4}\left(x_{4}-1\right)\left(1-r_{4}x_{4}\right)-y_{4}-g_{4}\left[\;\left(x_{4}-x_{3}\right)+\left(x_{4}-x_{1}\right)\right]\; & & \;\\ \;+I_{\mathrm{ext},4}+d_{\mathrm{ext},4},\\ {\dot{y}}_{4}=b_{4}x_{4}, \end{array}$$

where *x*_1_ and *y*_1_ are the model states of the master FHN neuron, namely, the activation potential and the recovery variable, respectively. The *x*_2_ and *y*_2_ represent the states of the first slave neuron, *x*_3_ and *y*_3_ correspond to the second slave neuron states, and *x*_4_ and *y*_4_ are the states for the fourth neuron. The parameters (*r*_1_, *r*_2_, *r*_3_, *r*_4_) and (*b*_1_, *b*_2_, *b*_3_, *b*_4_) are related with the neurons’ nonlinear parts and recovery variable dynamics, respectively. The terms *I_ext_*,_1_, *I_ext_*,_2_, *I_ext_*,_3_, and *I_ext_*,_4_ represent the external stimulation currents, where $I_{\mathrm{ext},i}=\left(A∕\omega \right)\cos\left(\omega t\right)$ for i=1,2,3,4, ω=2πf. Here, *f* denotes the frequency and *A* denotes the amplitude of stimulation current. The gap junctions’ strengths for communication between neurons are represented by *g*_1_, *g*_2_, *g*_3_, and *g*_4_. Disturbances at neurons are denoted by *d_ext_*,_1_, *d_ext_*,_2_, *d_ext_*,_3_, and *d_ext_*,_4_.

Various models of coupled neurons were considered in the studies (Wang et al., 2007, 2008a,b; Zhang et al., 2007; Che et al., 2009; Chen et al., 2009; Rehan and Hong, 2011; Rehan et al., 2011; Ambrosio and Aziz-Alaoui, 2012; Aqil et al., 2012a; Yu et al., 2012). However, these studies considered simple neuronal models with direction-independent coupling. The work of Iqbal et al. (2014) introduced the direction-dependent coupling. However, the ring configuration of neurons and coupling between several neurons were lacking. It should be noted that the model parameters associated with the proposed network of FHN neurons in Eqs (1)–(4) are totally uncertain and different. In addition, the proposed systematic approach considering direction-dependent coupling, different parameters, disturbances to the network model, and ring topology, in contrast to the simple models offered in Wang et al. (2007, 2008a,b), Zhang et al. (2007), Che et al. (2009), Chen et al. (2009), Rehan and Hong (2011), Rehan et al. (2011), Ambrosio and Aziz-Alaoui (2012), Aqil et al. (2012a), Yu et al. (2012), and Iqbal et al. (2014), which empowers a more realistic and generalized model.

**Figure 2:**
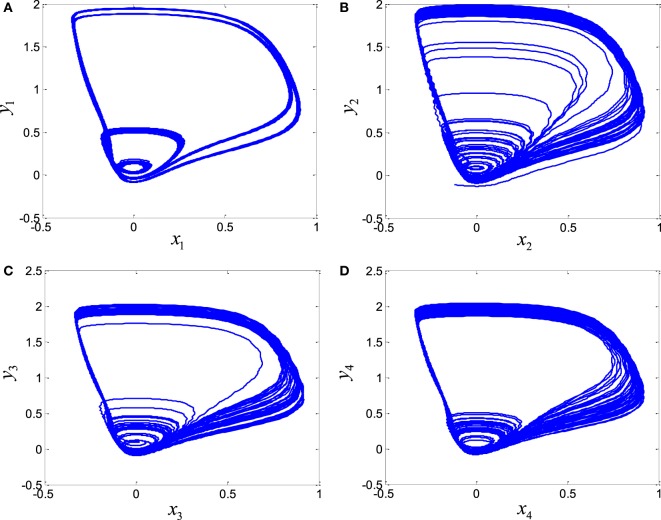
Chaotic behavior of four FitzHugh–Nagumo neurons without control: **(A)** first neuron, **(B)** second neuron, **(C)** third neuron, and **(D)** fourth neuron.

**Figure 3:**
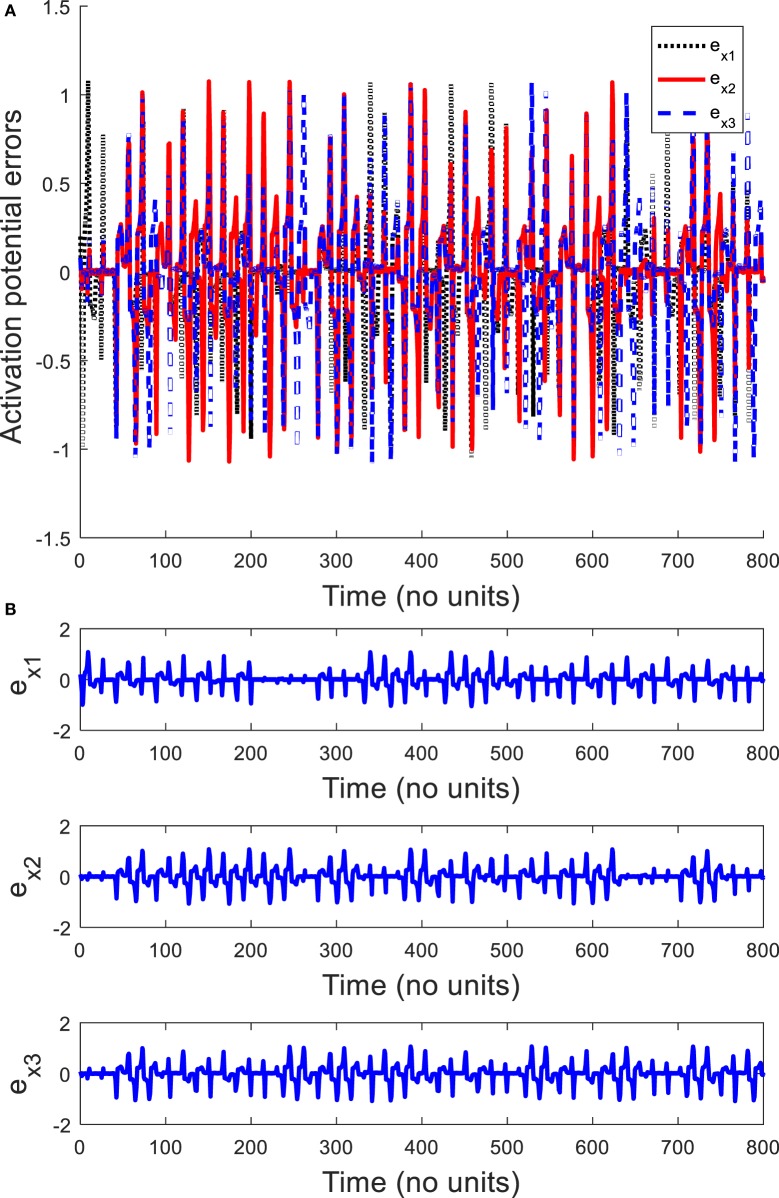
Activation potential errors in the absence of a control signal: **(A)** plots of activation potential errors, **(B)** spikes in activation potential errors *e_x_*_1_, *e_x_*_2_, _and_ *e_x_*_3_. It shows that all the activation potential errors have oscillating behaviors. Therefore, activation potentials of neurons are not synchronous.

**Figure 4:**
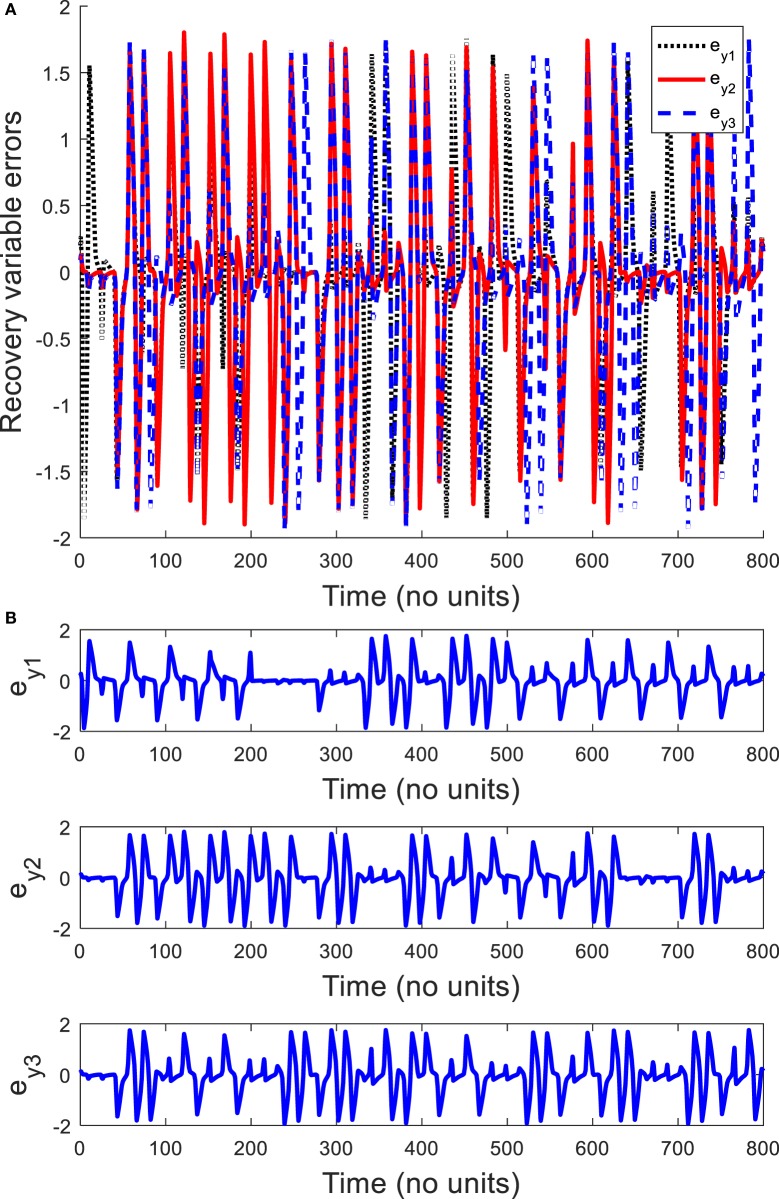
Recovery variable errors in the absence of a control signal: **(A)** plots of recovery variable errors, **(B)** spikes in recovery variable errors *e_y_*_1_, *e_y_*_2_,_and_ *e_y_*_3_. It shows that all the recovery variable errors have oscillatory behaviors. It can be concluded that recovery variables of FitzHugh–Nagumo neurons are not coherent.

In order to explore the complex behavior of the network model of the ring configured with different four FHN neurons under direction-dependent coupling, we first set the model parameters as *r*_1_ = 10, *r*_2_ = 10.2, *r*_3_ = 10.4, *r*_4_ = 10.6, *b*_1_ = 1, *b*_2_ = 1.01, *b*_3_ = 1.02, *b*_4_ = 1.03, *g*_1_ = 0.001, *g*_2_ = 0.002, *g*_3_ = 0.003, *g*_4_ = 0.004, and *f*  = 0.127. The disturbances are accounted as $d_{\mathrm{ext},1}=0.1\sin12t$, $d_{\mathrm{ext},2}=0.1\sin20t$, $d_{\mathrm{ext},3}=0.1\sin25t$, and $d_{\mathrm{ext},4}=0.1\sin23t$. The stimulation amplitude is selected as *A* = 0.01. Figure 2 depicts the results for the network of different FHN neurons under direction-dependent coupling. The phase portraits of four FHN chaotic neurons are shown in Figures 2A–D. These phase portraits show that the neurons have oscillatory behaviors. Figures 3 and 4 exhibit the nonsynchronous behavior of the network of four FHN neurons for activation potentials and recovery variables (to be explained later). The phase portrait in Figure 2A displays the chaotic behavior of first neuron. The second neuron’s chaotic behavior can be observed in Figure 2B. The chaotic behaviors for third and fourth neurons can be deduced from Figures 2C,D, respectively. The Lyapunov exponent has been computed for all the four neurons in Figures 2A–D using the approach provided in Iqbal et al. (2014), which come out to be 0.120, 0.058, 0.371, and 0.097. In conclusion, Figures 2–4 along with positive values of the Lyapunov exponent show that all of neurons in the network possess the chaotic behavior, as provided in Figures 2A–D, and are not synchronous, as indicated in Figures 3 and 4.

### Adaptive Control Mechanism and Error Dynamics

This section provides compact equations for the error dynamics, controller, and adaptation laws. This work offers an adaptive control mechanism for the synchronization of ring configured four FHN neurons under direction-dependent coupling. Thus, model in Eqs (1)–(4) becomes

(5) $$\begin{array}{lll} {\dot{x}}_{1}=x_{1}\left(x_{1}-1\right)\left(1-r_{1}x_{1}\right)-y_{1}-g_{1}\left[\;\left(x_{1}-x_{2}\right)+\left(x_{1}-x_{4}\right)\right]\; & & \;\\ \;+I_{\mathrm{ext},1}+d_{\mathrm{ext},1}, \end{array}$$

(6) $$\begin{array}{lll} {\dot{y}}_{1}=b_{1}x_{1},\; & \;\\ {\dot{x}}_{2}=x_{2}\left(x_{2}-1\right)\left(1-r_{2}x_{2}\right)-y_{2}-g_{2}\left[\;\left(x_{2}-x_{1}\right)+\left(x_{2}-x_{3}\right)\right]\; & & \;\\ \;+I_{\mathrm{ext},2}+d_{\mathrm{ext},2}+u_{x1}, \end{array}$$

(7) $$\begin{array}{lll} {\dot{y}}_{2}=b_{2}x_{2}+u_{y1},\; & & \;\\ {\dot{x}}_{3}=x_{3}\left(x_{3}-1\right)\left(1-r_{3}x_{3}\right)-y_{3}-g_{3}\left[\;\left(x_{3}-x_{2}\right)+\left(x_{3}-x_{4}\right)\right]\; & & \;\\ \;+I_{\mathrm{ext},3}+d_{\mathrm{ext},3}+u_{x2}, \end{array}$$

(8) $$\begin{array}{lll} {\dot{y}}_{3}=b_{3}x_{3}+u_{y2},\; & & \;\\ {\dot{x}}_{4}=x_{4}\left(x_{4}-1\right)\left(1-r_{4}x_{4}\right)-y_{4}-g_{4}\left[\;\left(x_{4}-x_{3}\right)+\left(x_{4}-x_{1}\right)\right]\; & & \;\\ \;+I_{\mathrm{ext},4}+d_{\mathrm{ext},4}+u_{x3},\\ {\dot{y}}_{4}=b_{4}x_{4}+u_{y3}, \end{array}$$

where $u_{x1}$, $u_{x2}$, and $u_{x3}$ and $u_{y1}$, $u_{y2}$, and $u_{y3}$ are the control inputs. We address a complete synchronization problem for the network model of ring configured FHN neurons in the context of their activation potentials and recovery variables, in contrast to the study of Iqbal et al. (2014), which has demonstrated the synchronization of two FHN neurons for their activation potentials only. To derive the control laws, the synchronization errors can be written as

(9) $$\begin{array}{l} e_{x1}=x_{1}-x_{2},e_{x2}=x_{1}-x_{3},e_{x3}=x_{1}-x_{4}, \end{array}$$

(10) $$\begin{array}{l} e_{y1}=y_{1}-y_{2},e_{y2}=y_{1}-y_{3},e_{y3}=y_{1}-y_{4}. \end{array}$$

It is worth mentioning that all six synchronization errors in Eqs (9) and (10) are introduced for attaining the complete synchronization, compared to the existing method of Iqbal et al. (2014). Figure 3A demonstrates the nonsynchronous behavior of neurons in terms of activation potentials. The spikes in the activation potential errors for the neurons can be observed in the plots of Figure 3B. On the same basis, demonstration of non-identical responses of the FHN neurons in the recovery variable states is provided in Figure 4A. The spikes in individual behaviors of synchronization errors in the recovery variables are provided in Figure 4B. These spikes in synchronization errors of activation potentials and recovery variables depict that the firing in neurons are not coherent at all. By employing Eqs (5)–(10), the synchronization error dynamics after lengthy algebra take the form

(11) $$\begin{array}{l} \begin{array}{l} {\dot{e}}_{x1}={\Phi_{1}}^{T}\Gamma_{1}\left(x_{1}\;,\;x_{2}\right)+F_{1}\left(x_{1}\;,\;x_{2}\right)-e_{x1}+d_{x1}-u_{x1},\\ {\dot{e}}_{y1}={\Psi_{1}}^{T}{ϒ}_{1}\left(x_{1}\;,\;x_{2}\right)-u_{y1}, \end{array} \end{array}$$

(12) $$\begin{array}{l} \begin{array}{l} {\dot{e}}_{x2}={\Phi_{2}}^{T}\Gamma_{2}\left(x_{1}\;,\;x_{3}\right)+F_{2}\left(x_{1}\;,\;x_{3}\right)-e_{x2}+d_{x2}-u_{x2},\\ {\dot{e}}_{y2}={\Psi_{2}}^{T}{ϒ}_{2}\left(x_{1}\;,\;x_{3}\right)-u_{y2}, \end{array} \end{array}$$

(13) $$\begin{array}{l} \begin{array}{l} {\dot{e}}_{x3}={\Phi_{3}}^{T}\Gamma_{3}\left(x_{1}\;,\;x_{4}\right)+F_{3}\left(x_{1}\;,\;x_{4}\right)-e_{x3}+d_{x3}-u_{x3},\\ {\dot{e}}_{y3}={\Psi_{3}}^{T}{ϒ}_{3}\left(x_{1}\;,\;x_{4}\right)-u_{y3}. \end{array} \end{array}$$

The whole derivation of the error dynamics and the relevant matrices can be seen in the Section “Methods”. The proposed controllers for the ring configured FHN neurons are selected as

(14) $$\begin{array}{l} \begin{array}{l} u_{x1}={{\hat{\Phi }}_{1}}^{T}\Gamma_{1}\left(x_{1}\;,\;x_{2}\right)+F_{1}\left(x_{1}\;,\;x_{2}\right)+K_{1}e_{x1},\\ u_{y1}={{\hat{\Psi }}_{1}}^{T}{ϒ}_{1}\left(x_{1}\;,\;x_{2}\right), \end{array} \end{array}$$

(15) $$\begin{array}{l} \begin{array}{l} u_{x2}={{\hat{\Phi }}_{2}}^{T}\Gamma_{2}\left(x_{1}\;,\;x_{3}\right)+F_{2}\left(x_{1}\;,\;x_{3}\right)+K_{2}e_{x2},\\ u_{y2}={{\hat{\Psi }}_{2}}^{T}{ϒ}_{2}\left(x_{1}\;,\;x_{3}\right), \end{array} \end{array}$$

(16) $$\begin{array}{l} \begin{array}{l} u_{x3}={{\hat{\Phi }}_{3}}^{T}\Gamma_{3}\left(x_{1}\;,\;x_{4}\right)+F_{3}\left(x_{1}\;,\;x_{4}\right)+K_{3}e_{x3},\\ u_{y3}={{\hat{\Psi }}_{3}}^{T}{ϒ}_{3}\left(x_{1}\;,\;x_{4}\right). \end{array} \end{array}$$

The selected adaptation laws are

(17) $$\begin{array}{l} \begin{array}{l} {\dot{\hat{\Phi }}}_{1}=p_{1}e_{x1}\Gamma_{1}\left(x_{1}\;,\;x_{2}\right)1∕q_{1},\\ {\dot{\hat{\Psi }}}_{1}=l_{1}e_{y1}{ϒ}_{1}\left(x_{1}\;,\;x_{2}\right)1∕m_{1}, \end{array} \end{array}$$

(18) $$\begin{array}{l} \begin{array}{l} {\dot{\hat{\Phi }}}_{2}=p_{2}e_{x2}\Gamma_{2}\left(x_{1}\;,\;x_{3}\right)1∕q_{2},\\ {\dot{\hat{\Psi }}}_{2}=l_{2}e_{y2}{ϒ}_{2}\left(x_{1}\;,\;x_{3}\right)1∕m_{2}, \end{array} \end{array}$$

(19) $$\begin{array}{l} \begin{array}{l} {\dot{\hat{\Phi }}}_{3}=p_{3}e_{x3}\Gamma_{3}\left(x_{1}\;,\;x_{4}\right)1∕q_{3},\\ {\dot{\hat{\Psi }}}_{3}=l_{3}e_{y3}{ϒ}_{3}\left(x_{1}\;,\;x_{4}\right)1∕m_{3}, \end{array} \end{array}$$

where the scalars sets (*p*_1_, *p*_2_, *p*_3_), (*q*_1_, *q*_2_, *q*_3_), (*l*_1_, *l*_2_, *l*_3_), and (*m*_1_,*m*_2_, *m*_3_) enclose positive scalars. In the next subsection, adaptive and robust adaptive synchronization control conditions are provided in the network of ring configured neurons.

### Adaptive Synchronization

Now, a theoretical condition is developed for the synchronization of ring configured neurons under direction-dependent coupling Eqs (5)–(8) by application of adaptive control mechanism in Eqs (14)–(16) with adaptation law in Eqs (17)–(19). The following assumption is taken to obtain the main results.

**Assumption 1**. The parameters in the network of four FHN neurons in Eqs (5)–(8) and couplings, given by ($r_{1},r_{2},r_{3},r_{4},b_{1},b_{2},b_{3},b_{4},g_{1},g_{2},g_{3},g_{4}$), are unknown constants.

**Theorem 1**.*Consider a network model of ring configured four FHN neurons in Eqs (5)–(8) having synchronization error dynamics Eqs (11)–(13) satisfying Assumption 1 with zero disturbances. Adaptive control mechanism Eqs (14)–(16) and the adaptation law given by Eqs (17)–(19) selected through* $p\left(K_{1}+1\right)>0$, $p\left(K_{2}+1\right)>0$, and $p\left(K_{3}+1\right)>0$*will ensure synchronization of the network model of ring configured neurons in terms of activation potentials by guaranteeing the convergence of synchronization errors to zero. In addition, if the steady-state is attained in a finite amount of time, the convergence of* ${\hat{\Phi }}_{i}$ *to* ${\hat{\Phi }}_{i}^{*}$*and* ${\hat{\Psi }}_{i}$ *to* ${\hat{\Psi }}_{i}^{*}$ *for all i* = *1, 2, 3, are ensured for constant steady-state vector values* ${\hat{\Phi }}_{i}^{*}$*and* ${\hat{\Psi }}_{i}^{*}$*, validating* ${\;\left(\Phi_{i}-{\hat{\Phi }}_{i}^{*}\right)}^{T}\Gamma_{i}=0$*and* ${\;\left(\Psi_{i}-{\hat{\Psi }}_{i}^{*}\right)}^{T}{ϒ}_{i}=0$.

The proof of the main result of Theorem 1 can be viewed in the next section. The result is important from the synchronization of a network of neurons point of view. In contrast to Iqbal et al. (2014), the proposed strategy in Theorem 1 can be used for complete synchronization of a network of different FHN neurons with unknown parameters. In addition, we considered multiple neurons linked in a ring configuration under direction-dependent coupling. In contrast to the conventional results like Wang et al. (2007, 2008a,b), Zhang et al. (2007), Che et al. (2009), Chen et al. (2009), Rehan and Hong (2011), Rehan et al. (2011), Ambrosio and Aziz-Alaoui (2012), Aqil et al. (2012a), and Yu et al. (2012), several aspects like uncertainties, ring configuration, different neurons, several number of neurons, and direction-dependent coupling are incorporated to design a matter-of-fact control approach of Theorem 1. Adaptations are employed for the synchronization of four neurons for dealing with a large number of unknown parameters. Additionally, a realistic approach has been followed for the adaptive control by considering all four neurons of different dynamics. The conventional studies assume that the FHN neurons have the same dynamical aspects.

In comparison to the works in Wang et al. (2007), Zhang et al. (2007), Wang et al. (2008a,b), Che et al. (2009), Rehan and Hong (2011), Lai et al. (2008), Wei et al. (2009), Che et al. (2011), Yu et al. (2012), and Ma et al. (2017), the proposed synchronization approach in Theorem 1 considers multiple neurons, directional coupling, and ring configuration to develop an adaptive mechanism for synchronization. The work of Rehan et al. (2011) considered synchronization in three neurons with known parameters. Here in this study, we consider adaptation of the parameters, and adaptation laws are introduced to achieve coherent behaviors in neurons with unknown and dissimilar parameters of neurons. In addition, a different configuration and direction-dependent couplings are employed in the proposed method of Theorem 1. The approach of Iqbal et al. (2015) developed a strategy to achieve synchronization in activation potentials and proposed a method to deal with two neurons only. In this case, we also provide a mechanism for synchronization recovery variables as well and provide an extension to a ring of four neurons.

### Robust Adaptive Synchronization with Disturbance

In this subsection, a methodology for the synchronization in a network of different FHN neurons with unknown parameters linked in a ring configuration under direction-dependent coupling and disturbances is presented. In addition to Assumption 1, we take the following supposition.

**Assumption 2**. Assume that the inequalities, given by $∥d_{x1}∥\leq d_{m1}$, $∥d_{x2}∥\leq d_{m2}$, $∥d_{x3}∥\leq d_{m3}$, and $∥\Phi_{i}∥\leq \Phi_{\mathrm{mi}},\forall i=1,2,3$, hold.

**Theorem 2**.*Consider a network model of ring configured four FHN neurons in Eqs (5)–(8), having synchronization error dynamics in Eqs (11)–(13) satisfying Assumptions 1–2. Suppose the proposed adaptive control mechanism in Eqs (14)–(16) and the modified adaptation laws given by*

(20) $$\begin{array}{l} \begin{array}{l} {\dot{\hat{\Phi }}}_{1}=\left(pe_{x1}\Gamma_{1}-k_{c}∥e_{x1}∥{\hat{\Phi }}_{1}\right)∕q\;,\\ {\dot{\hat{\Psi }}}_{1}=le_{y1}{ϒ}_{1}∕m, \end{array} \end{array}$$

(21) $$\begin{array}{l} \begin{array}{l} {\dot{\hat{\Phi }}}_{2}=\left(pe_{x2}\Gamma_{2}-k_{c}∥e_{x2}∥{\hat{\Phi }}_{2}\right)∕q\;,\\ {\dot{\hat{\Psi }}}_{2}=le_{y2}{ϒ}_{2}∕m, \end{array} \end{array}$$

(22) $$\begin{array}{l} \begin{array}{l} {\dot{\hat{\Phi }}}_{3}=\left(pe_{x3}\Gamma_{3}-k_{c}∥e_{x3}∥{\hat{\Phi }}_{3}\right)∕q\;,\\ {\dot{\hat{\Psi }}}_{3}=le_{y3}{ϒ}_{3}∕m, \end{array} \end{array}$$

*where k_c_ is a scalar constant. If we take* $p\left(K_{1}+1\right)>0$, $p\left(K_{2}+1\right)>0$, and $p\left(K_{3}+1\right)>0$*, it ensures synchronization of the network model of the ring configured FHN neurons by guaranteeing the convergence of errors to the compact sets. The proposed robust adaptive control scheme will ensure uniformly ultimately bounded errors and parameter estimation errors* $\Phi_{i}-{\hat{\Phi }}_{i}$.

A brief proof of the statement in Theorem 2 is presented in Section “Methods”. It is notable that the result of Theorem 2 refines the strategy developed in Theorem 1 by considering the disturbances to modify the design approach and adaptation laws. In contrast to the method demonstrated in Iqbal et al. (2014), the approach adopted in Theorem 2 provides a complete synchronization in a network of different FHN neurons with disturbance under unknown parameters linked in a ring configuration under direction-dependent coupling. There are various differences in this work with Iqbal et al. (2014). For instance, the four main differences are as follows: (a) we investigate a ring configuration of neurons, (b) this study is based on a more complex scenario of four neurons than the simple case of two neurons, (c) the coupling is also complex in this work, and (d) the achievement of complete synchronization rather than partial one is emphasized. It should also be noted that the work on synchronization of neurons under direction-dependent coupling is lacking in the literature. It is worth mentioning that such robust adaptive synchronization of the perturbed ring configured neurons with different parameters and direction-dependent coupling is lacking in the existing literature, like Wang et al. (2007, 2008a,b), Zhang et al. (2007), Che et al. (2009), Chen et al. (2009), Rehan and Hong (2011), Rehan et al. (2011), Ambrosio and Aziz-Alaoui (2012), Aqil et al. (2012a), and Yu et al. (2012). The presented approach considered a large number of parameters unknown in the four neurons. In addition, a perturbation in each neuron has been incorporated to provide an advanced synchronization solution. To deal with these perturbations and uncertainties, both adaptation and robustness of control signals for the slow and fast variations, respectively, are addressed in addition to the direction-dependent strength of the signals for any connection between neurons.

### Simulation Results

To validate the efficacy of the proposed adaptive control mechanism for synchronization in the network model of the ring configured different four FHN neurons under direction-dependent coupling, we first select the model parameters as *r*_1_ = 10, *r*_2_ = 10.2, *r*_3_ = 10.4, *r*_4_ = 10.6, *b*_1_ = 1, *b*_2_ = 1.01, *b*_3_ = 1.02, *b*_4_ = 1.03, *g*_1_ = 0.001, *g*_2_ = 0.002, *g*_3_ = 0.003, *g*_4_ = 0.004, and *f*  = 0.127. The disturbances are taken as $d_{\mathrm{ext},1}=0.1\sin12t$, $d_{\mathrm{ext},2}=0.1\sin20t$, $d_{\mathrm{ext},3}=0.1\sin25t$, and $d_{\mathrm{ext},4}=0.1\sin23t$. The stimulation amplitude is chosen as *A* = 0.01.

By application of Theorem 2, the parameters of controller and the adaptation law are obtained as *p* = *q* = *l* = *m* = 1. The control parameters are taken to be *k*_c_ = 5, *K*_1_ = 20, *K*_1_ = 20.001, and *K*_3_ = 20.002. It has been observed in Figures 2–4 that the behaviors of the original FHN neurons without any control signal are not coherent. As discussed earlier, the activation potential errors and recovery variable errors in Figures 3 and 4 do not have converging attributes. Rather, spikes are observed in the synchronization errors, leading to non-synchronous firings of the neurons.

**Figure 5:**
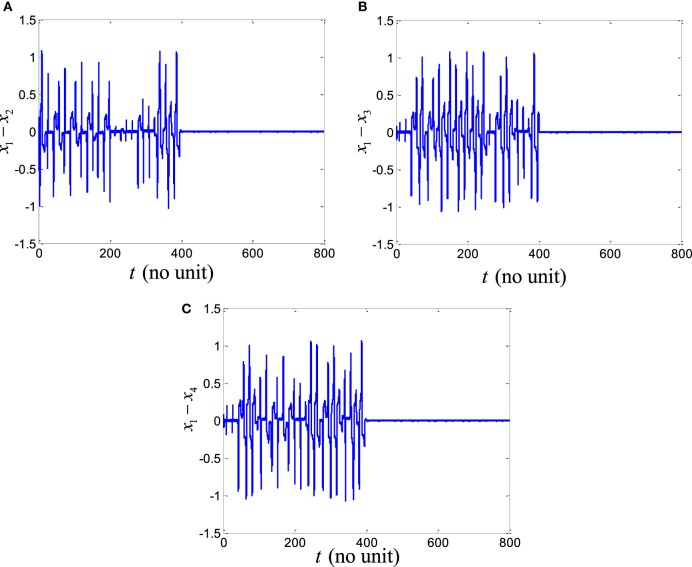
Activation potential errors of four FitzHugh–Nagumo neurons with the robust adaptive control in Eqs (14)–(16) and (20)–(22). The controller is applied at time *t* = 400. As controller is applied, synchronization of activation potentials is achieved: **(A)** error plot *x*_1_ − *x*_2_, **(B)** error plot *x*_1_ − *x*_3_, and **(C)** error plot *x*_1_ − *x*_4_.

**Figure 6:**
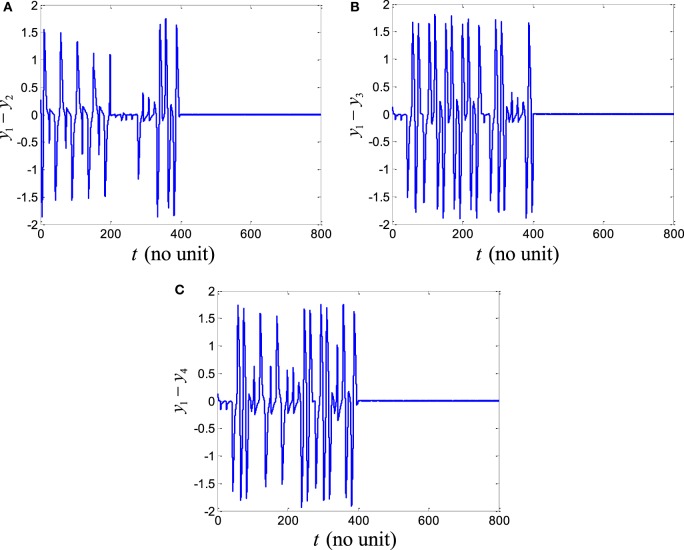
Recovery variable errors of four FitzHugh–Nagumo neurons under the robust adaptive control in Eqs (14)–(16) and (20)–(22). The controller is applied at time *t* = 400. As controller is applied, synchronization of recovery variable is achieved: **(A)** error plot *y*_1_ − *y*_2_, **(B)** error plot *y*_1_ − *y*_3_, and **(C)** error plot *y*_1_ − *y*_4_.

Now we simulate the behavior of same neurons without and with the proposed robust adaptive control scheme of Theorem 2. The proposed control signal is applied at *t* = 400. Before this time, the behaviors of the neurons are not coherent and the synchronization errors have oscillatory responses. By means of the proposed robust adaptive control scheme, it is observed that the FHN neurons are synchronized under unknown parameters and external perturbations. Figures 5 and 6 depict the synchronization errors for the different FHN neurons under direction-dependent coupling by using the proposed methodology. Before *t* = 400, the behaviors of the activation potential errors in Figure 5 have spikes, showing non-synchronous firing in neurons. The same trend is also observed in the recovery variable synchronization errors in Figure 6. We activated the proposed robust adaptive controller of Theorem 2 at *t* = 400. By application of the controller, the synchronization errors for activation potentials and recovery variables converge to a region near zero, as shown in Figures 5 and 6. The convergence of synchronization errors is fast, showing the effectiveness of the proposed robust adaptive control scheme. Due to convergence of the synchronization errors in Figures 5 and 6, the spikes due to firing of the four neurons under bidirectional coupling become identical, validating the synchronization in both activation potentials and recovery variables. It is concluded that the results in Figure 5 authenticate the efficacy of the proposed robust adaptive control mechanism in the context of synchronization of activation potentials. Moreover, Figure 6 validates the effectiveness of the proposed mechanism for synchronization of recovery variables. As the synchronization errors converge in the neighborhood of zero, it is evident that synchronization of activation and recovery potentials is achieved *via* the proposed robust adaptive control scheme.

The adopted modeling and control methodologies are generalized in certain extent and simulation results presented herein represent a broader scenario of a network of FHN neurons. The methods presented in Theorems 1–2 are valid to a general form of FHN neurons. In addition, robustness against bounded disturbances has been guaranteed through Theorem 2. The results of Theorems 1 and 2 may not be limited to FHN systems of only four neurons. All in all, the proposed modeling and control methodology can be used for a more general form, synchronization in a network of different FHN neurons of unknown parameters, coupled in ring configuration, and subject to direction-dependent coupling and disturbances.

## Materials and Methods

### FHN Model

Neuron is the chief functional element in the brain. Its dynamical examination is important for the treatment of brain diseases. There are many neuronal models, such as Hindmarsh-Rose, Hodgkin and Huxley, and FitzHugh–Nagumo, etc. These models offer investigation of the dynamical behavior of a neuron and even synchronization in a network of neurons. FHN model is a famous one in terms of representing various neuronal behaviors, owing to its simple representation. Consider the FHN model for representing dynamical aspects of a neuron subjected to external electrical stimulation as in Thompson et al. (1999), given by

(23) $$\begin{array}{l} \begin{array}{l} \frac{\mathrm{dx}}{\mathrm{dt}}=x\left(x-1\right)\left(1-\mathrm{rx}\right)-y+I,\\ \frac{\mathrm{dy}}{\mathrm{dt}}=\mathrm{bx}+\mathrm{vy}, \end{array} \end{array}$$

where *x* and *y* represent the activation potential and the recovery variable, respectively, *r* is a nonlinearity parameter in the model, parameters *b* and *v* are related to the recovery variable, and I=(a∕ω) cos ωt shows the stimulation current. We employ this important neuronal model to study the synchronization in a network of different FHN neurons of unknown parameters coupled in ring configuration subject to direction-dependent coupling and disturbances. In this paper, coupled FHN models were simulated using the *S*-function in Matlab for nonlinear differential equations.

### Lyapunov Stability Analysis

The Lyapunov stability criterion is widely utilized to understand the stability and control of dynamical systems. In order to elaborate the Lyapunov stability method, consider a dynamical system, for example, ẋ=f(t,x), where $x\in R^{n}$ denotes the state vector for the dynamical system. Suppose there exists a positive definite Lyapunov function *V* (*x*) for all the values of vector $x\in R^{n}$. If the derivative of the energy function *V* (*x*) along the dynamics of the system *x* = *f* (*t, x*) is negative definite, the state *x* will approach to zero, conferring to the Lyapunov stability theory (see Khalil (1996) and references therein). $\dot{V}\left(x\right)<0$ means that the factitious energy *V* (*x*) of the dynamical system is decreasing, leading to stability of the system.

### Derivation of Error Dynamics

By using Eqs (5)–(10), we obtain the error dynamics as follows:

(24) $$\begin{array}{ll} {ė}_{x1}=f_{1}\left(x_{1}\right)-f_{2}\left(x_{2}\right)-y_{1}+y_{2}-g_{1}\left[\;\left(x_{1}-x_{2}\right)+\left(x_{1}-x_{4}\right)\right] & \\ \;+g_{2}\left[\;\left(x_{2}-x_{1}\right)+\left(x_{2}-x_{3}\right)\right]+d_{x1}-u_{x1},\\ {ė}_{y1}=b_{1}x_{1}-b_{2}x_{2}-u_{y1}, \end{array}$$

(25) $$\begin{array}{l} {ė}_{x2}=f_{1}\left(x_{1}\right)-f_{3}\left(x_{3}\right)-y_{1}+y_{3}-g_{1}\left[\;\left(x_{1}-x_{2}\right)+\left(x_{1}-x_{4}\right)\right]\\ \;+g_{3}\left[\;\left(x_{3}-x_{2}\right)+\left(x_{3}-x_{4}\right)\right]+d_{x2}-u_{x2},\\ {ė}_{y2}=b_{1}x_{1}-b_{3}x_{3}-u_{y2}, \end{array}$$

(26) $$\begin{array}{l} {ė}_{x3}=f_{1}\left(x_{1}\right)-f_{4}\left(x_{4}\right)-y_{1}+y_{4}-g_{1}\left[\;\left(x_{1}-x_{2}\right)+\left(x_{1}-x_{4}\right)\right]\\ \;+g_{4}\left[\;\left(x_{4}-x_{3}\right)+\left(x_{4}-x_{1}\right)\right]+d_{x3}-u_{x3},\\ {ė}_{y3}=b_{1}x_{1}-b_{4}x_{4}-u_{y3}. \end{array}$$

Note that *I_ext_*_,1_, *I_ext_*_,2_, *I_ext_*_,3_, and *I_ext_*_,4_ are the same in the present scenario, therefore, their effect is canceled out in the error dynamics. Let us define the functions and signals

(27) $$\begin{array}{l} \begin{array}{l} f_{1}\left(x_{1}\right)=-r_{1}x_{1}^{3}+r_{1}x_{1}^{2}+x_{1}^{2}-x_{1},\\ f_{2}\left(x_{2}\right)=-r_{2}x_{2}^{3}+r_{2}x_{2}^{2}+x_{2}^{2}-x_{2},\\ f_{3}\left(x_{3}\right)=-r_{3}x_{3}^{3}+r_{3}x_{3}^{2}+x_{3}^{2}-x_{3},\\ f_{4}\left(x_{4}\right)=-r_{4}x_{4}^{3}+r_{4}x_{4}^{2}+x_{4}^{2}-x_{4},\\ \;d_{x1}=d_{\mathrm{ext},1}-d_{\mathrm{ext},2},\\ \;d_{x2}=d_{\mathrm{ext},1}-d_{\mathrm{ext},3},\\ \;d_{x3}=d_{\mathrm{ext},1}-d_{\mathrm{ext},4}. \end{array} \end{array}$$

As the recovery variable dynamics are dependent on the activation potential, the relations become

(28) $$\begin{array}{l} \begin{array}{r} y_{1}=b_{1}\int_{0}^{t}x_{1}d\alpha +y_{1}\left(0\right),\\ y_{2}=b_{2}\int_{0}^{t}x_{2}d\alpha +y_{2}\left(0\right),\\ y_{3}=b_{3}\int_{0}^{t}x_{3}d\alpha +y_{3}\left(0\right),\\ y_{4}=b_{4}\int_{0}^{t}x_{4}d\alpha +y_{4}\left(0\right). \end{array} \end{array}$$

Here *y*_1_(0), *y*_2_(0), *y*_3_(0), and *y*_4_(0) denote the unknown initial conditions for the recovery variable of four neurons. The relevant quantities in the error dynamics formulation are defined by

(29) $$\begin{array}{l} \begin{array}{rl} {\Phi_{1}}^{T} & =\left[\begin{array}{cccccccc} r_{1} & r_{2} & b_{1} & b_{2} & y_{1}\left(0\right) & y_{2}\left(0\right) & g_{1} & g_{2} \end{array}\right]\\ \Gamma_{1}\left(x_{1},\;x_{2}\right) & =\left[\begin{array}{cccccc} -x_{1}^{3}+x_{1}^{2} & x_{2}^{3}-x_{2}^{2} & -\int_{0}^{t}x_{1}d\alpha & \int_{0}^{t}x_{2}d\alpha & -1 & 1, \end{array}\right.\\ & \;-{\left.\begin{array}{cc} \left[\;\left(x_{1}\;-\;x_{2}\right)\;+\;\left(x_{1}\;-\;x_{4}\right)\right] & \left[\;\left(x_{2}\;-\;x_{1}\right)\;+\;\left(x_{2}\;-\;x_{3}\right)\right] \end{array}\right]}^{T}, \end{array} \end{array}$$

(30) $$\begin{array}{l} \begin{array}{rl} {\Psi_{1}}^{T} & =\left[\begin{array}{cc} b_{1} & b_{2} \end{array}\right]\;,\left[0,1\right]\;,\\ {ϒ}_{1}\left(x_{1},\;x_{2}\right) & ={\left[\begin{array}{cc} x_{1} & -x_{2} \end{array}\right]}^{T}, \end{array} \end{array}$$

(31) $$\begin{array}{l} \begin{array}{rl} {\Phi_{2}}^{T} & =\left[\begin{array}{cccccccc} r_{1} & r_{3} & b_{1} & b_{3} & y_{1}\left(0\right) & y_{3}\left(0\right) & g_{1} & g_{3} \end{array}\right]\;,\\ \Gamma_{2}\left(x_{1},x_{3}\right) & =\left[\begin{array}{cccccc} -x_{1}^{3}+x_{1}^{2} & x_{3}^{3}-x_{3}^{2} & -\int_{0}^{t}x_{1}d\alpha & \int_{0}^{t}x_{3}d\alpha & -1 & 1, \end{array}\right.\\ & \;{\left.\begin{array}{cc} -\left[\;\left(x_{1}\;-\;x_{2}\right)\;+\;\left(x_{1}\;-\;x_{4}\right)\right] & \left[\;\left(x_{3}\;-\;x_{2}\right)\;+\;\left(x_{3}\;-\;x_{4}\right)\right] \end{array}\right]}^{T}, \end{array} \end{array}$$

(32) $$\begin{array}{l} \begin{array}{rl} {\Psi_{2}}^{T} & =\left[\begin{array}{cc} b_{1} & b_{3} \end{array}\right],\\ {ϒ}_{2}\left(x_{1},x_{3}\right) & ={\left[\begin{array}{cc} x_{1} & -x_{3} \end{array}\right]}^{T}, \end{array} \end{array}$$

(33) $$\begin{array}{l} \begin{array}{rl} {\Phi_{3}}^{T} & =\left[\begin{array}{cccccccc} r_{1} & r_{4} & b_{1} & b_{4} & y_{1}\left(0\right) & y_{4}\left(0\right) & g_{1} & g_{4} \end{array}\right]\;,\\ \Gamma_{3}\left(x_{1},x_{4}\right) & =\left[\begin{array}{cccccc} -x_{1}^{3}+x_{1}^{2} & x_{4}^{3}-x_{4}^{2} & -\int_{0}^{t}x_{1}d\alpha & \int_{0}^{t}x_{4}d\alpha & -1 & 1, \end{array}\right.\\ & \;{\left.\begin{array}{cc} -\left[\;\left(x_{1}\;-\;x_{2}\right)\;+\;\left(x_{1}-x_{4}\right)\right] & \left[\;\left(x_{4}\;-\;x_{3}\right)\;+\;\left(x_{4}\;-\;x_{1}\right)\right] \end{array}\right]}^{T}, \end{array} \end{array}$$

(34) $$\begin{array}{l} \begin{array}{rl} {\Psi_{3}}^{T} & =\left[\begin{array}{cc} b_{1} & b_{4} \end{array}\right],\\ {ϒ}_{3}\left(x_{1},x_{4}\right) & ={\left[\begin{array}{cc} x_{1} & -x_{4} \end{array}\right]}^{T}, \end{array} \end{array}$$

and

(35) $$\begin{array}{l} \begin{array}{rl} & F_{1}\left(x_{1},x_{2}\right)=x_{1}^{2}-x_{2}^{2},\\ & F_{2}\left(x_{1},x_{3}\right)=x_{1}^{2}-x_{3}^{2},\\ & F_{3}\left(x_{1},x_{4}\right)=x_{1}^{2}-x_{4}^{2}. \end{array} \end{array}$$

Employing Eqs (27)–(35) into Eqs (24)–(26), the error dynamics equations given by Eqs (11)–(13) are obtained in the Section “Results and Discussion”.

### Proof of Theorem 1

The proof of Theorem 1 is provided using the same steps as in Iqbal et al. (2014). However, our scenario is more complex due to the ring configuration and multiple neurons. Incorporating Eqs (14)–(16) into Eqs (11)–(13), for *i* = 1, 2, 3 leads to the results

(36) $$\begin{array}{l} \begin{array}{l} {ė}_{\mathrm{xi}}={\;\left(\Phi_{i}-{\hat{\Phi }}_{i}\right)}^{T}\Gamma_{i}-\left(K_{i}+1\right)e_{\mathrm{xi}}+d_{\mathrm{xi}},\\ {ė}_{\mathrm{yi}}={\;\left(\Psi_{i}-{\hat{\Psi }}_{i}\right)}^{T}{ϒ}_{i}. \end{array} \end{array}$$

The considered Lyapunov function candidate is given by

(37) $$\begin{array}{l} \begin{array}{rl} & V\left(e_{\mathrm{xi}},e_{\mathrm{yi}},\left(\Phi_{i}-{\hat{\Phi }}_{i}\right),\left(\Psi_{i}-{\hat{\Psi }}_{i}\right)\right)\\ & \;=\left(1∕2\right)\sum_{i=1}^{3}\left(p{e_{\mathrm{xi}}}^{2}+q{\;\left(\Phi_{i}-{\hat{\Phi }}_{i}\right)}^{T}\left(\Phi_{i}-{\hat{\Phi }}_{i}\right)\right)\\ & \;+\left(1∕2\;\right)\sum_{i=1}^{3}\left(l{e_{\mathrm{yi}}}^{2}+m{\;\left(\Psi_{i}-{\hat{\Psi }}_{i}\right)}^{T}\left(\Psi_{i}-{\hat{\Psi }}_{i}\right)\right), \end{array} \end{array}$$

with *p* > 0, *q* > 0, *l* > 0, *m* > 0. On taking the time-derivative of Eq (37), using ${\;\left(\Phi_{i}-{\hat{\Phi }}_{i}\right)}^{T}{\dot{\hat{\Phi }}}_{i}\;=\;{{\dot{\hat{\Phi }}}_{i}}^{T}\left(\Phi_{i}-{\hat{\Phi }}_{i}\right)$ and ${\;\left(\Psi_{i}-{\hat{\Psi }}_{i}\right)}^{T}{\dot{\hat{\Psi }}}_{i}={\dot{\hat{\Psi }}}_{i}{\;\left(\Psi_{i}-{\hat{\Psi }}_{i}\right)}^{T}$ and, further, incorporating the error systems of Eq (36), we obtain

(38) $$\begin{array}{l} \begin{array}{rl} & \dot{V}\left(e_{\mathrm{xi}},e_{\mathrm{yi}},\left(\Phi_{i}-{\hat{\Phi }}_{i}\right),\left(\Psi_{i}-{\hat{\Psi }}_{i}\right)\right)\\ & \;=\sum_{i=1}^{3}\left(pe_{\mathrm{xi}}{\;\left(\Phi_{i}-{\hat{\Phi }}_{i}\right)}^{T}\Gamma_{i}-p\left(K_{i}+1\right){e_{\mathrm{xi}}}^{2}\right.\\ & \;-q{\;\left(\Phi_{i}-{\hat{\Phi }}_{i}\right)}^{T}{\dot{\hat{\Phi }}}_{i}+pe_{\mathrm{xi}}d_{\mathrm{xi}}+le_{\mathrm{yi}}{\;\left(\Psi_{i}-{\hat{\Psi }}_{i}\right)}^{T}{ϒ}_{i}\\ & \;\left.-m{\;\left(\Psi_{i}-{\hat{\Psi }}_{i}\right)}^{T}{\dot{\hat{\Psi }}}_{i}\right). \end{array} \end{array}$$

Using the adaptation laws in Eqs (17)–(19) under zero disturbances, it yields

(39) $$\dot{V}\left(e_{\mathrm{xi}},e_{\mathrm{yi}},\left(\Phi_{i}-{\hat{\Phi }}_{i}\right),\left(\Psi_{i}-{\hat{\Psi }}_{i}\right)\right)=-p\sum_{i=1}^{3}\left(K_{i}+1\right){e_{\mathrm{xi}}}^{2}.$$

As $\dot{V}\left(e_{\mathrm{xi}},e_{\mathrm{yi}},\left(\Phi_{i}-{\hat{\Phi }}_{i}\right),\left(\Psi_{i}-{\hat{\Psi }}_{i}\right)\right)<0$, we need $-p\left(K_{i}+1\right)$ less than zero for i=1,2,3. In the steady-state, the synchronization errors and their derivatives are zero. In addition, the behaviors of all four neurons will be the same. Therefore, we have ${\dot{\hat{\Phi }}}_{i}=0$ and ${\dot{\hat{\Psi }}}_{i}=0$, which implies that ${\hat{\Phi }}_{i}={{\hat{\Phi }}^{*}}_{i}$ and ${\hat{\Psi }}_{i}={{\hat{\Psi }}^{*}}_{i}$ are satisfied in the steady-state, where ${{\hat{\Phi }}^{*}}_{i}$ and ${{\hat{\Psi }}^{*}}_{i}$ are constants. As observed in Rehan and Hong (2011), Rehan et al. (2011), and Iqbal et al. (2014), we have ${\;\left(\Phi_{i}-{{\hat{\Phi }}^{*}}_{i}\right)}^{T}\Gamma_{i}=0$ and ${\;\left(\Psi_{i}-{{\hat{\Psi }}^{*}}_{i}\right)}^{T}{ϒ}_{i}=0$.

### Proof of Theorem 2

The proof of Theorem 2 employs similar methods as in the results (Rehan and Hong, 2011; Rehan et al., 2011; Iqbal et al., 2014) for the proposed complex scenario. Using Eq (38) and the proposed adaptation law in Theorem 2, we have

(40) $$\begin{array}{l} \begin{array}{rl} & \dot{V}\left(e_{\mathrm{xi}},e_{\mathrm{yi}},\left(\Phi_{i}-{\hat{\Phi }}_{i}\right),\left(\Psi_{i}-{\hat{\Psi }}_{i}\right)\right)\\ & \;=\sum_{i=1}^{3}\left(-p\left(K_{i}+1\right){e_{\mathrm{xi}}}^{2}-{\;\left({\hat{\Phi }}_{i}-\Phi_{i}\right)}^{T}{\hat{\Phi }}_{i}k_{c}∥e_{\mathrm{xi}}∥+pe_{\mathrm{xi}}d_{\mathrm{xi}}\right). \end{array} \end{array}$$

It can be confirmed with $∥\Phi_{i}∥\leq \Phi_{\mathrm{mi}}$ that ${∥{\hat{\Phi }}_{i}-\Phi_{i}∥}^{2}-∥{\hat{\Phi }}_{i}-\Phi_{i}∥\Phi_{\mathrm{mi}}\leq {\;\left({\hat{\Phi }}_{i}-\Phi_{i}\right)}^{T}{\hat{\Phi }}_{i}$ from (Iqbal et al., 2014). It along with Assumption 2 implies

(41) $$\begin{array}{l} \begin{array}{l} \dot{V}\left(e_{\mathrm{xi}},e_{\mathrm{yi}},\left(\Phi_{i}-{\hat{\Phi }}_{i}\right),\left(\Psi_{i}-{\hat{\Psi }}_{i}\right)\right)\\ \;\;\leq \sum_{i=1}^{3}\left(-∥e_{\mathrm{xi}}∥\left(p\left(K_{i}+1\right)∥e_{\mathrm{xi}}∥+k_{c}{\left(∥{\hat{\Phi }}_{i}-\Phi_{i}∥-\Phi_{\mathrm{mi}}∕2\right)}^{2}\right.\right.\\ \;\left.\left.-k_{c}\Phi_{\mathrm{mi}}^{2}∕4-pd_{\mathrm{mi}}\right)\right). \end{array} \end{array}$$

Given that *p*(*K_i_ * +  1) > 0, Eq (41) implies that $\dot{V}\left(e_{\mathrm{xi}},e_{\mathrm{yi}},\left(\Phi_{i}\;-\;{\hat{\Phi }}_{i}\right),\;\left(\Psi_{i}-{\hat{\Psi }}_{i}\right)\right)<0$ if the conditions in Eq (42) hold.

(42) $$∥e_{\mathrm{xi}}∥>\frac{k_{c}\Phi_{\mathrm{mi}}^{2}∕4\;+pd_{\mathrm{mi}}}{p\left(K_{i}+1\right)},∥{\hat{\Phi }}_{i}-\Phi_{i}∥>\frac{\Phi_{\mathrm{mi}}}{2}+\sqrt{\frac{\Phi_{\mathrm{mi}}^{2}}{4}+\frac{pd_{\mathrm{mi}}}{k_{c}}},$$

for *i* = 1, 2, 3. Thus, the synchronization errors and estimation errors are uniformly ultimately bounded as seen in Zhang et al. (2007), Rehan and Hong (2011), Rehan et al. (2011), and Iqbal et al. (2014) and references therein. The guidelines provided in Zhang et al. (2007), Rehan and Hong (2011), Rehan et al. (2011), and Iqbal et al. (2014) and references therein for the selections of robust adaptive control parameters can be followed.

This study provides a step to increase complexity by increasing the number of neurons and considering their complex interactions, and it provides an approach to consider a generalized model for synchronization aspects. Prohibition of synchronization is also another research topic. Further works on blockage of the synchronization using control strategies can also be investigated.

## Conclusions

This paper addressed the controlled synchronization in a network of ring configured four different FHN neurons with unknown parameters under direction-dependent coupling and disturbances. The neurons and their interactions (i.e., coupling) in a ring topology network are considered to be different owing to the inter-neuronal coupling medium properties. Based on the Lyapunov stability criteria, adaptive control strategies were developed to deal with the complex problem of synchronization in a network of four different FHN neurons. In addition, a robust adaptive control was also developed to ensure robustness against the external disturbances to attain the uniformly ultimately bounded synchronization errors. In contrast to various existing works, dissimilar neurons, unknown parameters, multiple neurons, ring topology of neurons, bidirectional communication in neurons and coherence in activation potentials, and recovery variables are incorporated in this study. The numerical simulation results verified the efficacy of the proposed control approaches.

## Author Contributions

MI wrote the first draft of the manuscript. MR has initiated the idea and revised the manuscript. K-SH has corrected the manuscript and finalized the work. All the authors have approved the final manuscript.

## Conflict of Interest Statement

The authors declare that the research was conducted in the absence of any commercial or financial relationships that could be construed as a potential conflict of interest.
